# Doença cística adventicial da artéria poplítea: relato de caso

**DOI:** 10.1590/1677-5449.009217

**Published:** 2018

**Authors:** Julio Cesar Peclat de Oliveira, Fernando Tebet Ramos Barreto, Diogo Di Battista de Abreu e Souza, João Marcos Fonseca e Fonseca, Bernardo de Castro Abi Ramia Chimelli, Ana Paula Rolim Maia Peclat, Marcos Arêas Marques, Stenio Karlos Alvim Fiorelli

**Affiliations:** 1 Clínica Julio Peclat, Cirurgia Vascular, Rio de Janeiro, RJ, Brasil.; 2 Hospital Municipal Miguel Couto, Cirurgia Vascular, Rio de Janeiro, RJ, Brasil.; 3 Clínica Julio Peclat, Clínica Médica, Rio de Janeiro, RJ, Brasil.; 4 Universidade do Estado do Rio de Janeiro – UERJ, Angiologia, Rio de Janeiro, RJ, Brasil.

**Keywords:** artéria poplítea, claudicação intermitente, enxerto vascular

## Abstract

A doença cística adventicial da artéria poplítea é uma doença pouco frequente, que deve ser considerada no diagnóstico diferencial de pacientes jovens com claudicação intermitente e sem fatores de risco para doença arterial periférica aterosclerótica. Apresentamos um caso de claudicação intermitente de membros inferiores em paciente masculino de 51 anos no qual essa doença foi diagnosticada. Foi submetido a ressecção do segmento de artéria comprometido e interposição de safena autóloga ipsilateral. Discutimos alternativas diagnósticas e terapêuticas.

## INTRODUÇÃO

A doença cística adventicial (DCA) é uma doença rara, caracterizada pela formação de cistos de conteúdo mucinoso na camada adventícia de veias ou artérias, de etiologia desconhecida e descrita pela primeira vez em 1947^1^. O crescimento do cisto pode ocasionar redução do lúmen do vaso e, em associação ao fato de que a artéria poplítea é o vaso mais acometido, justifica o principal sintoma apresentado pelos pacientes ser a claudicação intermitente (CI) de pernas, em indivíduos de meia idade sem fatores de risco para doença aterosclerótica^2^^,^ 3.

Apresentamos o caso de um paciente sem fatores de risco para doença arterial periférica (DAP) de origem aterosclerótica, com CI de perna esquerda ocasionada por DCA da artéria poplítea.

## DESCRIÇÃO DO CASO

Paciente do sexo masculino, 51 anos de idade, praticante de atividade física regular (corredor de meia-maratona), sem fatores de risco para DAP aterosclerótica e sem patologias previamente conhecidas. Queixou-se de CI de membro inferior esquerdo para distâncias de aproximadamente 100 metros, iniciada havia oito meses, com piora progressiva que limitava o desempenho de suas atividades diárias regulares, sem outras queixas associadas no período. De relevância na história familiar, havia um irmão falecido por aneurisma de aorta abdominal roto, aos 53 anos.

Ao exame físico, observava-se diminuição da amplitude dos pulsos poplíteo e podais à esquerda, em comparação com o membro contralateral. Não havia diferenças significativas de temperatura ou coloração entre os membros. O índice tornozelo-braquial (ITB) em repouso, aferido nas artérias pediosas, era de 0,98 à direita e 0,68 à esquerda.

**Figura 1:**
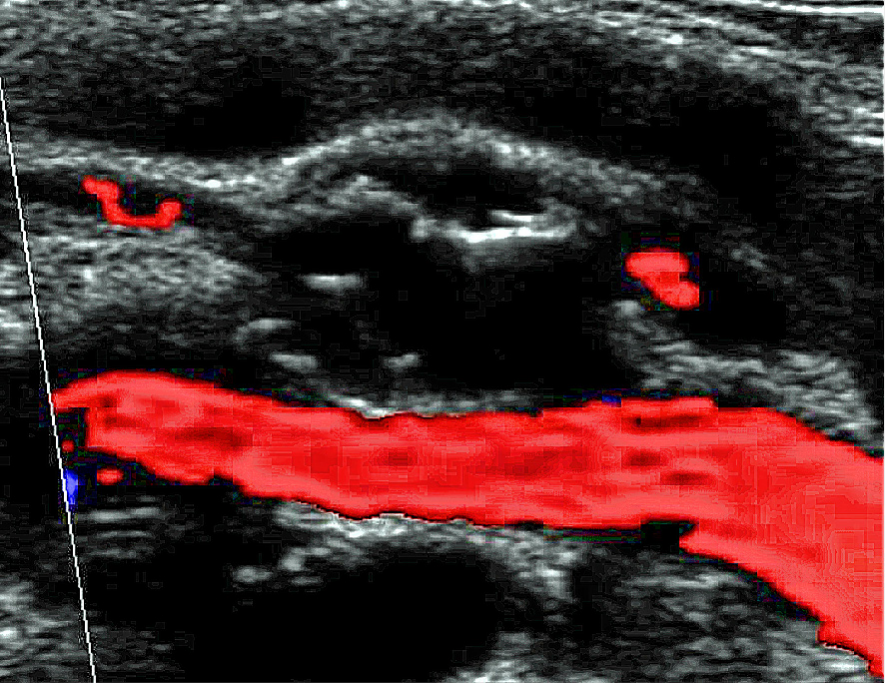
Eco-Doppler colorido arterial demostrando lesão cística septada na camada adventícia da artéria poplítea esquerda, com compressão extrínseca e discreta redução do calibre do vaso.

**Figura 2:**
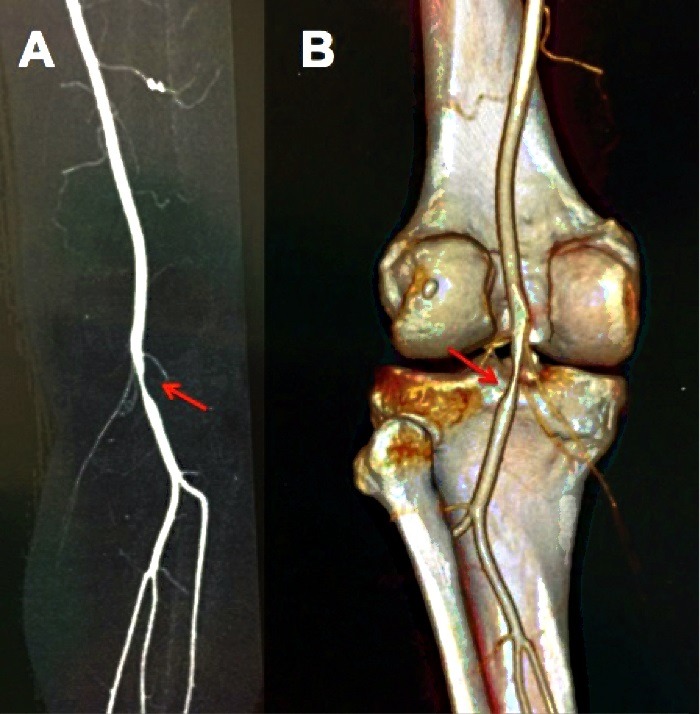
Reconstruções de angiotomografia evidenciando a estenose ocasionada pelo cisto em exposição anterior (A) e posterior (B).

Foi solicitado eco-Doppler colorido (EDC) arterial de membros inferiores, que demonstrou a presença de lesões císticas de paredes finas e bem definidas adjacentes à artéria poplítea esquerda, causando compressão extrínseca da artéria (Figura 1). Não havia lesões nos outros segmentos arteriais estudados nesse membro, nem no membro contralateral. Prosseguiu-se a investigação com angiotomografia computadorizada (ATC) de membros inferiores, que corroborou a presença de imagem cística, assim como a estenose ocasionada na artéria poplítea esquerda (Figuras 2A e 2B).

**Figura 3:**
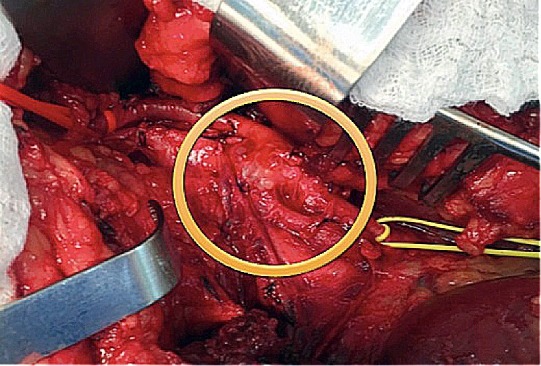
Segmento da artéria poplítea esquerda com múltiplos cistos de conteúdo mucinoso na camada adventícia.

**Figura 4:**
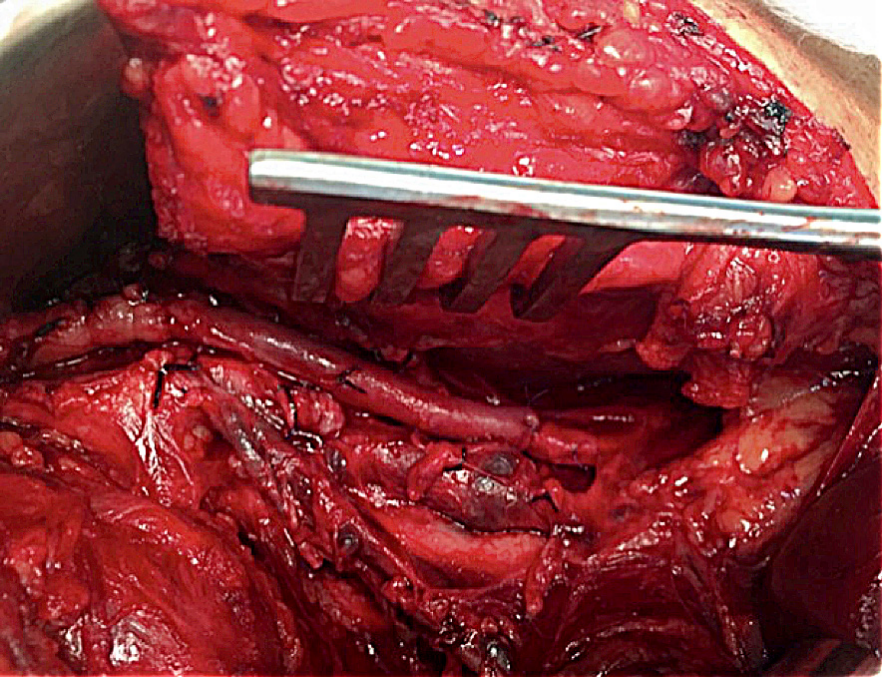
Após a ressecção do segmento afetado, foi realizada interposição de enxerto com veia safena reversa ipsilateral.

Como a CI provocava grande limitação funcional ao paciente, foi indicado tratamento cirúrgico convencional com ressecção do segmento arterial acometido e interposição com enxerto de veia safena autóloga reversa ipsilateral (Figuras 3, 4 e 5). O segmento arterial ressecado foi enviado para estudo anatomopatológico, que confirmou a presença de cisto mucinoso na camada adventícia da artéria poplítea.

**Figura 5:**
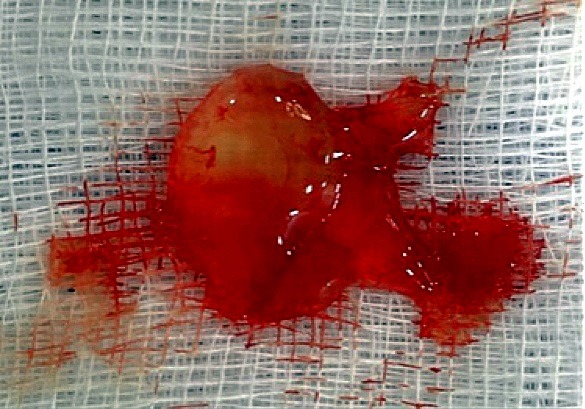
Cisto ressecado e isolado da artéria poplítea esquerda. Observa-se que apresenta paredes muito finas e conteúdo claro.

O período pós-operatório transcorreu sem intercorrências, com aumento da amplitude dos pulsos podais em relação ao período anterior ao procedimento. Houve, ainda, aumento do ITB, que passou a ser de 0,91 no membro inferior esquerdo.

A alta hospitalar ocorreu 48 horas após a cirurgia. Em um mês, foi realizado novo EDC arterial do membro inferior esquerdo, que evidenciou fluxos normais, ausência de estenoses e curvas trifásicas em todos os segmentos estudados. Após dois anos do procedimento, o paciente mantém-se assintomático, tendo retomado por completo todas as atividades que desempenhava antes do surgimento dos sintomas. Faz acompanhamento com EDC arterial anual, que, até o momento, demonstra bom resultado cirúrgico.

## DISCUSSÃO

A DCA é uma doença rara e responde por um a cada 1.200 casos de CI de membros inferiores^4^. A artéria poplítea é o vaso mais acometido, mas há relato de doença em outros seguimentos arteriais e veias. Diferentemente dos aneurismas de artéria poplítea, o acometimento bilateral é extremamente raro. Há inúmeras teorias para o surgimento da doença, mas nenhuma se demonstrou definitiva até o momento.

A apresentação clínica clássica é a de um homem na quarta ou quinta década de vida com CI progressiva. Deve-se aumentar ainda mais o grau de suspeição se o paciente não apresenta fatores de risco clássicos para DAP de origem aterosclerótica^5^^-^ 7. O exame físico pode ser normal, mas pode ocorrer a redução ou abolição dos pulsos distais à flexão do joelho (sinal de Ishikawa)^8^.

A manifestação da doença se dá em consequência da compressão extrínseca da artéria acometida pelo cisto, podendo variar desde CI apenas em atividades extenuantes até oclusão arterial aguda, que pode ocorrer em até 30% dos casos^9^. A resolução espontânea do quadro clínico pode ocorrer, mas geralmente há reincidência da doença^10^.

Os exames de imagem são essenciais para confirmação do diagnóstico, sendo o EDC arterial o primeiro a ser solicitado na investigação da doença. Brodmann et al. sugere que esse é o método de imagem mais sensível para comprovação da DCA^11^ e que a presença de uma imagem compatível com lesão cística (imagem hipoecoica ou anecoica) adjacente à artéria, causando sua compressão, associada aos sintomas do paciente, é suficiente para indicar o tratamento cirúrgico.

**Figura 6:**
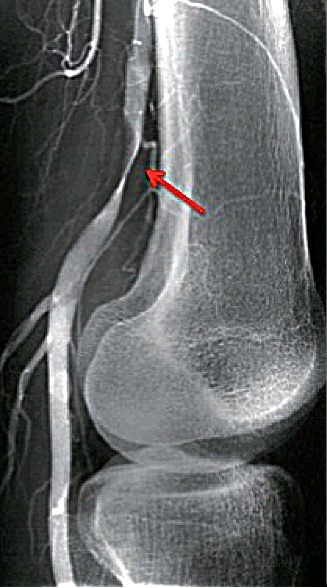
Angiografia demonstrando o sinal da cimitarra, um afilamento alongado e curvilíneo da luz da artéria poplítea (seta).

A ressonância nuclear magnética (RNM) e a ATC apresentam melhor definição anatômica da região poplítea e devem ser realizadas para um adequado planejamento cirúrgico. A realização de angiografia como exame complementar é questionável por se tratar de um método invasivo e que pode não definir o diagnóstico, visto que o exame pode ser normal e, na ausência de estenoses, não é possível confirmar a presença do cisto, pois a angiografia só evidencia dados sobre a luz das artérias, e não sobre a sua parede. No entanto, a presença do sinal da cimitarra (Figura 6) na angiografia sugere o diagnóstico^6^^,^ 9.

O tratamento está indicado sempre que a doença é limitante para o paciente. Em casos com pouco ou nenhum sintoma, cabe avaliar o risco e o benefício da realização do procedimento cirúrgico, visando impedir a progressão da estenose arterial e a sua potencial oclusão futura. As opções terapêuticas incluem: aspiração percutânea do cisto sob visão ultrassonográfica ou tomográfica, angioplastia percutânea com ou sem implante de *stent*, enucleação do cisto com manutenção das camadas média e íntima da artéria e ressecção segmentar da artéria acometida pela doença seguida de interposição com enxerto venoso^12^^,^ 13.

A aspiração do cisto pode não ser possível por dificuldades em se conseguir uma janela para acessá-lo ou pela alta viscosidade do seu conteúdo. Além disso, a aspiração apresenta altos índices de recorrência, visto que as células responsáveis pela formação dos cistos são mantidas^14^.

O tratamento com angioplastia transluminal percutânea também não apresenta bons resultados, devido à reestenose por aumento da pressão extrínseca causada pelo cisto que não foi ressecado. O implante de *stents* em segmento de artéria poplítea, especialmente em pacientes jovens, é ainda questionável^13^^,^ 15.

Os melhores resultados são obtidos com a ressecção cirúrgica do cisto e da adventícia acometida, seja com manutenção ou ressecção segmentar da artéria^7^^,^ 16. A ressecção com interposição de enxerto é recomendada principalmente nos casos em que há oclusão arterial.

## CONCLUSÃO

A DCA da artéria poplítea é rara e deve ser suspeitada em casos de CI de membros inferiores em pacientes jovens e com baixo ou nenhum risco para DAP aterosclerótica. Por apresentar poucos sinais sugestivos ao exame físico, os exames de imagem são essenciais para o diagnóstico, sendo o EDC o exame mais sensível. A RNM e a ATC devem ser realizadas após a confirmação diagnóstica pelo EDC, para o planejamento terapêutico. O tratamento cirúrgico está indicado para todos os pacientes com limitação importante de suas atividades diárias. Os demais pacientes terão a indicação baseada em riscos e benefícios em longo prazo. A terapia de escolha é a ressecção do segmento arterial acometido e a interposição com veia autóloga, preferencialmente a safena magna ipsilateral reversa. Entendemos que essa é a terapia que confere maior certeza de ressecção de todo o tecido doente.

Por se tratar de uma doença rara, a literatura ainda é escassa e estudos com maiores números de participantes e com maior tempo de acompanhamento se fazem necessários para eliminar dúvidas existentes acerca da doença.
