# Supplementary material for: Doença cística adventicial da artéria poplítea: relato de caso
Source: J Vasc Bras. 2018 Jan-Mar;17(1):55–60. [Article in Portuguese] doi: 10.1590/1677-5449.009217 (PMC5990258; doi:10.1590/1677-5449.009217)
Supplement: Translation

# Translation: Adventitial cystic disease of the popliteal artery: case report

**Authors:** Julio Cesar Peclat de Oliveira, Fernando Tebet Ramos Barreto, Diogo Di Battista de Abreu e Souza, João Marcos Fonseca e Fonseca, Bernardo de Castro Abi Ramia Chimelli, Ana Paula Rolim Maia Peclat, Marcos Arêas Marques, Stenio Karlos Alvim Fiorelli

**Affiliations:** 1 Clínica Julio Peclat, Cirurgia Vascular, Rio de Janeiro, RJ, Brasil.; 2 Hospital Municipal Miguel Couto, Cirurgia Vascular, Rio de Janeiro, RJ, Brasil.; 3 Clínica Julio Peclat, Clínica Médica, Rio de Janeiro, RJ, Brasil.; 4 Universidade do Estado do Rio de Janeiro (UERJ), Angiologia, Rio de Janeiro, RJ, Brasil.

**Keywords:** popliteal artery, intermittent claudication, vascular grafting

## Abstract

Adventitial cystic disease of the popliteal artery is an uncommon pathology that should be considered in differential diagnostic of younger patients with intermittent claudication and without risk factors for peripheral atherosclerotic arterial disease. We report the case of a 51 year-old male patient presenting with lower-limb intermittent claudication in whom this pathology was diagnosed and who was treated with segmental arterial resection and autologous saphenous vein interposition. We also discuss diagnostic and therapeutic alternatives.

## INTRODUCTION

Adventitial cystic disease (ACD) is a rare disease, characterized by formation of cysts containing mucin in the adventitial layer of veins or arteries, with unknown etiology, that was described for the first time in 1947.^1^ Growth of the cyst can reduce the lumen of the vessel which, in combination with the fact that the popliteal artery is the vessel most often affected, explains why the principal symptom exhibited by patients is intermittent claudication (IC) of the leg, in middle-aged people without risk factors for atherosclerotic disease.^2^^,^ 3

We present the case of a patient without risk factors for peripheral arterial disease (PAD) of atherosclerotic origin, with IC of the left leg caused by ACD involving the popliteal artery.

## CASE DESCRIPTION

The patient was a 51-year-old male, who regularly practiced physical activity (a half-marathon runner), without risk factors for atherosclerotic PAD and with no prior known pathologies. He complained of IC in the left lower limb after walking distances of approximately 100 meters, with onset 8 months previously and progressive deterioration, and limiting his ability to perform regular daily activities, with no other associated complaints during this period. Of relevance in his family history was a brother who had died from a ruptured abdominal aortic aneurysm at the age of 53 years.

On physical examination, the amplitudes of the left popliteal and pedal pulses were found to be smaller than those in the contralateral limb. There were no significant differences in temperature or color between the two limbs. The ankle-brachial indices (ABI) for the pedal arteries at rest were 0.98 on the right and 0.68 on the left.

Color Doppler ultrasonography (CDUS) of the arterial system of the lower limbs showed cystic lesions with thin, well-defined walls adjacent to the left popliteal artery, causing extrinsic compression of the artery (Figure 1). There were no lesions in other arterial segments studied in this limb or in the contralateral limb. Investigation was continued using computed tomography angiography (CTA) of the lower limbs, which confirmed the presence of cystic images and consequent stenosis of the left popliteal artery (Figures 2A and 2B).

**Figure 1:** Color Doppler ultrasonography, showing walled cystic lesion in the adventitial layer of the left popliteal artery, with extrinsic compression and discrete reduction in the caliber of the vessel.

**Figure 2:** Reconstructed angiotomography images showing stenosis caused by the cyst in anterior (A) and posterior (B) views.

Since IC causes major functional limitation to patients, conventional surgical treatment was prescribed, with resection of the affected arterial segment and interposition of a graft constructed from the ipsilateral autologous saphenous vein, reversed (Figures 3, 4, and 5). The resected arterial segment was sent for anatomopathological investigation, which confirmed the presence of mucin cysts in the adventitial layer of the popliteal artery.

**Figure 3:** Segment of the left popliteal artery with multiple cysts containing mucin in the adventitial layer.

**Figure 4:** After resection of the affected segment, interposition of a reverse ipsilateral saphenous vein graft.

**Figure 5:** Cyst resected and isolated from the left popliteal artery. Observe the very thin walls and pale content.

There were no intercurrent conditions during the postoperative period and the pedal pulse amplitude increased in comparison to the preoperative measurement. The ABI also increased, reaching 0.91 in the left lower limb.

The patient was discharged from hospital 48 hours after surgery. One month later, arterial CDUS of the left lower limb was performed, once more, showing normal flows, absence of stenosis, and three-phase curves in all segments studied. Two years after the procedure, the patient is still asymptomatic and has completely resumed all of the activities he performed before onset of symptoms. He attends for annual follow-up arterial CDUS, which has shown good surgical results to date.

## DISCUSSION

Adventitial cystic disease is a rare disease that is responsible for one in every 1,200 cases of lower limb IC.^4^ The vessel most often involved is the popliteal artery, but there are reports of the disease in other segments of arteries and in veins. In contrast to popliteal artery aneurysms, bilateral ACD is extremely rare. There are countless theories to explain emergence of the disease, but none have been proven the definitive cause to date.

The classic clinical presentation is a male in his fourth or fifth year of life with progressive IC. The level of suspicion is increased if he does not have the classic risk factors for PAD of atherosclerotic origin.^5^^-^ 7 Physical examination may be normal, but there may be a reduction or total absence of distal pulses when the knee is in flexion (Ishikawa’s sign).^8^

The manifestations of the disease are a consequence of extrinsic compression of the artery involved by the cyst and can range from IC only during strenuous activities to acute arterial occlusion, which can occur in up to 30% of cases.^9^ Spontaneous resolution of clinical status may be observed, but the disease generally recurs.^10^

Imaging exams are essential for confirmation of the diagnosis, and arterial CDUS is the first option for investigation of the disease. Brodmann et al. suggest that this is the most sensitive imaging method for confirmation of ACD^11^ and that presence of an image compatible with a cystic lesion (a hypoechoic or anechoic image) adjacent to the artery, causing compression, in conjunction with the patient’s symptoms, is sufficient indication for surgical treatment.

Magnetic resonance imaging (MRI) and CTA offer better anatomic definition in the popliteal area and should be used to adequately plan surgery. Use of angiography as a supplementary examination is questionable, because it is an invasive method and may not confirm diagnosis. Findings may be normal and, in the absence of stenosis, it is not capable of confirming that a cyst is present, because angiography only provides evidence related to the lumen of arteries, and not their walls. Notwithstanding, the scimitar sign (Figure 6) on angiography is suggestive of a diagnosis of ACD.^6^^,^ 9

**Figure 6:** Angiography showing the scimitar sign, a long, curved, narrowing of the popliteal artery lumen (arrowed).

Treatment is indicated whenever the disease is limiting for the patient. In cases with few or no symptoms, it is advisable to consider the risks and benefits of performing the surgical procedure with the objective of impeding progression of arterial stenosis and its potential occlusion in the future. Treatment options include percutaneous aspiration of the cyst, guided by ultrasonography or tomography, percutaneous angioplasty with or without stent placement, enucleation of the cyst with preservation of the medial and intimal layers of the artery, and segmental resection of the artery affected by the disease followed by interposition of a venous graft.^12^^,^ 13

Aspiration of the cyst may not be possible because of difficulties obtaining a window to access it or because of the high viscosity of its contents. Furthermore, aspiration is linked with high rates of recurrence, since the cells responsible for formation of the cysts are still present.^14^

The results of treatment with percutaneous transluminal angioplasty are also poor, since it is associated with restenosis caused by increased extrinsic pressure from the cyst that is not resected. Additionally, placement of stents in segments of the popliteal artery is still questionable, especially in younger patients.^13^^,^ 15

The best results are achieved with surgical resection of the cyst and of the adventitial tissue involved, with maintenance or segmental resection of the artery.^7^^,^ 16 Resection with graft interposition is primarily recommended in cases in which there is arterial occlusion.

## CONCLUSIONS

Adventitial cystic disease of the popliteal artery is rare, but should be suspected in cases of lower limb IC in young patients with little or no risk of atherosclerotic PAD. There are few suggestive signs that can be detected by physical examination, so imaging exams are essential for diagnosis; among which CDUS is the most sensitive examination. Magnetic resonance imaging and CTA should be performed after diagnosis has been confirmed by CDUS, to plan treatment. Surgical treatment is indicated for all patients with significant limitations of daily activities. Other patients should be managed on the basis of risks and benefits over the long term. The treatment of choice is resection of the affected arterial segment and interposition of an autologous vein graft, preferably the ipsilateral great saphenous, reversed. We consider that this is the treatment that offers the greatest certainty that all of the diseased tissue has been resected.

Since this is a rare disease, there is still scant literature and studies with larger patient numbers and longer follow-ups are needed to eliminate doubts that still remain about this disease.
